# Review of Supported Pd-Based Membranes Preparation by Electroless Plating for Ultra-Pure Hydrogen Production

**DOI:** 10.3390/membranes8010005

**Published:** 2018-01-23

**Authors:** David Alique, David Martinez-Diaz, Raul Sanz, Jose A. Calles

**Affiliations:** Department of Chemical and Energy Technology, Rey Juan Carlos University, C/Tulipán s/n, Móstoles, 28933 Madrid, Spain; david.alique@urjc.es (D.A.); david.martinez.diaz@urjc.es (D.M.-D.); raul.sanz@urjc.es (R.S.)

**Keywords:** review, palladium, membrane, Pd alloy, electroless plating, membrane reactor, hydrogen separation, hydrogen production

## Abstract

In the last years, hydrogen has been considered as a promising energy vector for the oncoming modification of the current energy sector, mainly based on fossil fuels. Hydrogen can be produced from water with no significant pollutant emissions but in the nearest future its production from different hydrocarbon raw materials by thermochemical processes seems to be more feasible. In any case, a mixture of gaseous compounds containing hydrogen is produced, so a further purification step is needed to purify the hydrogen up to required levels accordingly to the final application, i.e., PEM fuel cells. In this mean, membrane technology is one of the available separation options, providing an efficient solution at reasonable cost. Particularly, dense palladium-based membranes have been proposed as an ideal chance in hydrogen purification due to the nearly complete hydrogen selectivity (ideally 100%), high thermal stability and mechanical resistance. Moreover, these membranes can be used in a membrane reactor, offering the possibility to combine both the chemical reaction for hydrogen production and the purification step in a unique device. There are many papers in the literature regarding the preparation of Pd-based membranes, trying to improve the properties of these materials in terms of permeability, thermal and mechanical resistance, poisoning and cost-efficiency. In this review, the most relevant advances in the preparation of supported Pd-based membranes for hydrogen production in recent years are presented. The work is mainly focused in the incorporation of the hydrogen selective layer (palladium or palladium-based alloy) by the electroless plating, since it is one of the most promising alternatives for a real industrial application of these membranes. The information is organized in different sections including: (i) a general introduction; (ii) raw commercial and modified membrane supports; (iii) metal deposition insights by electroless-plating; (iv) trends in preparation of Pd-based alloys, and, finally; (v) some essential concluding remarks in addition to futures perspectives.

## 1. Introduction

The continuous population growth and economy intensification imply an increase of global energy demand. Up to date, this increasing demand has been usually covered by massive use of fossil fuels, causing global warming due to the large emission of anthropogenic greenhouse gases, as well as other combustion pollutants [1]. This situation is even more problematic because the fossil resources worldwide depletion and thus, it represents a clearly unsustainable scenario for the future. In the last years, it has been suggested a wide set of alternatives for the progressive replacement of fossil fuels as primary energy resource [2,3]. In this situation, the implementation of the so-called hydrogen-based economy is considered as a real choice and it is receiving great attention in the last years [4,5,6,7,8]. 

Hydrogen is advised as a very promising energy carrier due to its long-term viability, high energy density (14 J·kg^−1^·°C^−1^), environmentally welcoming combustion emissions and high resources to be produced from. Indeed, hydrogen is the most abundant element in the Earth, although it is usually found combined with other elements, mainly in water and hydrocarbon molecules. The idea is to transfer the energy obtained for different primary energy sources, preferentially renewables (i.e., wind, solar or biomass, among others), to hydrogen, which can be stored, transported and eventually used in different energy applications. Ideally, hydrogen will be obtained from water by using these renewable energies, thus minimizing the environmental impact while the energy demand is covered [9,10]. However, the hydrogen generation by thermochemical processes seems to be a more realistic option in the near future for cost-cutting [11,12,13,14]. Hence, hydrogen can be generated from a wide variety of raw materials containing hydrocarbons for both centralized and distributed production systems by using relatively mature technologies [14], being the use of biomass and waste materials especially attractive [15,16,17,18,19]. In these cases, a mixture of gaseous compounds is frequently produced, being necessary to purify the hydrogen up to required levels accordingly to the final application, i.e., PEM fuel cells, turbines or combustion engines [20]. Indeed, the hydrogen purification step is a crucial process in the successful implementation of the hydrogen energy system from both technical and economical point of view. 

**Figure 1:**
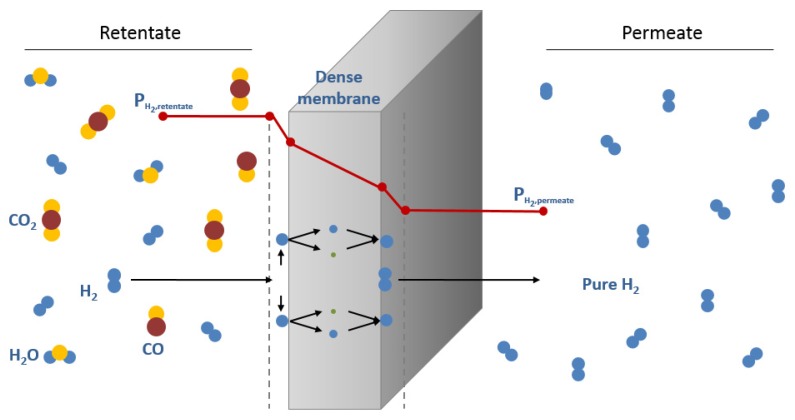
Solution-diffusion mechanism for hydrogen permeation through the metal lattice of a dense membrane.

Among readily available alternatives for hydrogen purification, the use of membranes for hydrogen separation/production applications has been proposed and used in practice. This technology shows relevant advantages such as low energy consumption, environmentally good properties and also additional potential to be combined with a reaction unit in a multifunctional membrane reactor [21,22]. The combination of simultaneous chemical reaction and hydrogen separation in one unique step results in additional benefits in terms of conversion increase by shifting the reaction equilibrium as one of the products, hydrogen, is selectively separated from the reaction media [23,24]. Particularly, dense metallic based membranes have been proposed for years due to their potential to transport hydrogen in a dissociative form with a theoretically complete perm-selectivity [25,26]. Thus, the structure of metals belonged groups III-V, such as Pd, Ni and Pt (in pure form and alloyed), has the ability to allow the hydrogen diffusion through the metal lattice, while avoiding the permeation of other molecules [27,28]. In this way, the solution-diffusion mechanism, depicted in Figure 1, is used to describe the hydrogen permeation process in these H_2_-selective membranes.

**Figure 2:**
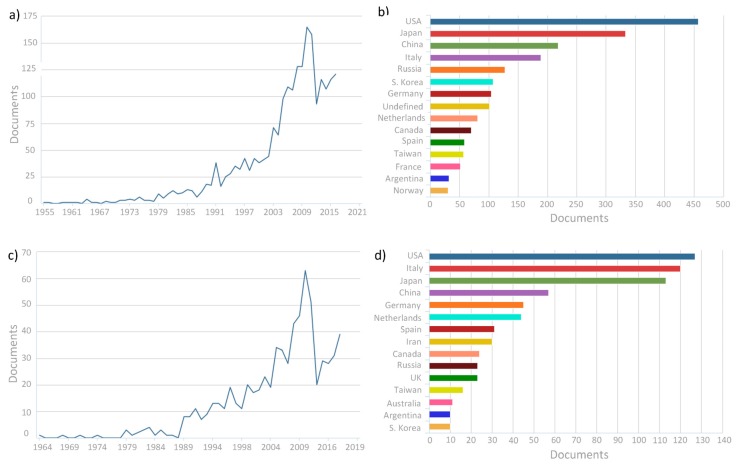
Citation analysis report by Scopus for keywords: palladium + membrane + hydrogen (**a**,**b**) and palladium + membrane reactor + hydrogen (**c**,**d**).

Up to date, palladium is the most studied metal for preparing these H_2_-selective membranes. The earliest studies date from the XIX century, when Deville and Troost discovered the capability of hydrogen to penetrate into bulk palladium [29,30] and Graham determined that this metal was able to absorb hundred times its own volume in hydrogen [31]. However, the use of palladium membranes for hydrogen separation/production applications does not appear until the fifties years. From this decade, as the related research publications evidence, these membranes have been gaining increasing interest. This trend can be observed in Figure 2, where it is shown the number of scientific documents published by year and region considering hydrogen, palladium and membrane or membrane reactor as keywords. It has to be pointed out the increase of related publications during the most recent years, mainly due to a greater awareness on environmental protection and renewable energies development, where hydrogen emerges as a very promising clean energy vector that, as mentioned before, requires to be purified [20,25]. 

Analysing the number of publications by region, it is evident that this topic is investigated widely around the word, topping the list the United States of America with very ambitious policies but closely followed by different countries of Asia (Japan and China) and Europe (mainly Italy, Germany and The Netherlands).

Currently, main efforts are focused to reduce the cost of these membranes and to increase its mechanical resistance, lifespan and fabrication reproducibility [32,33]. Palladium is expensive and scarce and the growing demand for its use in large-scale applications is expected to keep driving up its price [34]. Two of the most studied strategies to reduce the cost of the membranes are: (a) minimizing the amount of palladium required to achieve a fully dense layer [35,36,37,38] and (b) increasing the use life-span since these membranes can suffer deactivation by poisoning and cracking by thermal or mechanical stress [39,40,41,42,43]. Taking into account the typical equation used to describe the hydrogen permeation flux ($J_{H_{2}}$) through a Pd-based membrane (Richardson equation, Equation (1)) as function of hydrogen permeability (*k*), metal thickness (*t*) and pressure driving force $\left(P_{H_{2},ret}^{n}-P_{H_{2},per}^{n}\right)$, it is obvious that a decrease in the metal thickness provokes an increase of the permeation capability [28,44].

(1) $$J_{H_{2}}=\frac{k}{t}\left(P_{H_{2},ret}^{n}-P_{H_{2},per}^{n}\right)$$

In case of the Pd-based membrane is totally free of defects, the hydrogen permeation is determined by the solution-diffusion in the bulk metal and the exponential factor takes the value *n* = 0.5, denoting the equation as Sieverts’ law.

However, the preparation of ultrathin palladium layers entails two main problems: (i) limitation of membrane mechanical resistance and (ii) difficulty to obtain films free of defects. The use of porous supports tries to overcome these problems and thus, to maintain adequate mechanical properties saving palladium [45,46,47,48,49].

On the other hand, many authors focus their efforts in developing new fabrication processes for ensuring a better reproducibility and reducing the number of rejected membranes [49,50,51] or modifying the selective layer (Pd-based alloys) in order to improve some particular properties, such as resistance to hydrogen embrittlement or deactivation by sulphur compounds [52,53,54,55,56].

Several technologies can be used to incorporate a thin film of the hydrogen selective metal, preferentially Pd or Pd-based alloy, onto a porous support. Cold-rolling [57,58,59], physical vapour deposition [60,61,62,63], chemical vapour deposition [64,65,66], electrochemical plating [67,68,69] and electroless plating can be mentioned [33,62,70,71]. The last option (electroless plating, or its acronym ELP) provides important advantages in terms of adherence and uniformity of deposits on both conducting and non-conducting surfaces with complex geometries. Additionally, it has manufacturing low cost, becoming very popular for most of the studies carried out in the literature [72,73,74,75]. 

Considering all these facts, this review expects to provide a general overview of the most recent and relevant advances for preparation of dense Pd-based membranes for hydrogen production in membrane reactors, particularly focused on supported membranes obtained by electroless plating technology onto inorganic porous supports. The manuscript is divided into different sections focused on: (i) materials, pre-treatments and surface modifications of raw membrane supports, (ii) palladium deposition by electroless plating and (iii) development of new metal Pd-based alloys. Finally, some essential concluding remarks and brief comments about trendy futures perspectives have been also included.

## 2. Membrane Supports

Dense Pd-based membranes can be classified in two main groups, unsupported and supported ones, in which a thin selective film is deposited onto a porous substrate. The first type is usually prepared from relatively thick palladium (or Pd-based alloys) foils that, as Tosti et al. indicate in case of requiring tubular geometry, are cold-rolled and welded [17,76]. Typical thicknesses are ranged from 50 to 150 μm. However, as previously mentioned, a thick Pd layer strongly hinders both hydrogen permeate rate and membrane cost. Thus, development of new ultrathin membranes without jeopardizing mechanical resistance and presence of defects is the main objective of many researchers in this field [59,60,77]. This goal is usually achieved by incorporating a thin Pd layer on the surface of a porous material that provides the required mechanical resistance to the supported membrane [71,78,79,80]. This complex task is subject of numerous studies since many factors must be considered, i.e., the compatibility between support and selective layer, which strongly determines the mechanical resistance of the membrane due to cracks can be formed at high temperatures because of different expansion coefficients, as it will be discussed in detail later. 

Numerous porous materials, such as Vycor glass [81,82], sintered metals [71,78,83], a wide variety of ceramics [53,71,84,85] and even polymers [86,87,88], can be used as supporting materials for the H_2_-selective layer. The most relevant attributes of supports to be selected include porosity properties (mainly average porosity and pore sizes distribution), surface roughness and mechanical, chemical and thermal stabilities [89]. In this context, it is expected great porosity with a narrow distribution of small pore sizes, high mechanical strength and chemical resistance and similar thermal expansion coefficient to that of Pd [90]. In regards of textural properties, the support porosity needs to be open and interconnected enough to ensure a non-limiting gas transport through the support, besides the critical sizes of pore-mouth and of pore-throat [91]. It is accepted that usually both pore size and roughness strongly determine morphology and continuity of the selective layer. In this mean, Mardilovich et al. [92] indicated that the minimum thickness necessary to prepare an electroless plated Pd film onto a porous support is around three times the average size of the greatest pores. Despite Vycor glass was one of the first porous supports used to incorporate Pd by electroless plating [81,82], currently is more frequent the use of sintered porous metals [71,78,93,94] or ceramic materials [53,71,84,95], making a clean sweep on the majority of scientific publications in this field. 

The use of porous polymers as substrates for membranes reactors, which usually operates a high temperature, is currently scarce due to the low thermal resistance of these materials [96]. For this particular application, the metallic supports are the preferred ones, such as stainless-steel 316L [71,83], Hastelloy [97,98], Inconel [94], nickel [99] or, in some particular cases, Ti-based alloys such as Ti-Al [100] or Ni-Ti [101]. They usually ensure good mechanical properties, hardness and adequate thermal expansion coefficient, similar to that of palladium, in the range of 10.5–12.5 × 10^−6^ °C^−1^. Moreover, these materials are easily sealed and coupled to membrane reactor modules, conventionally made of stainless-steel [96]. However, these supports present relatively large pores with a wide pore sizes distribution that makes the generation of a thin and free-defect Pd layer difficult. In fact, it is usual that manufactures do not provide the concrete value of pore sizes in these supports, giving an average related value, known as media grade, that represents the particle size that is rejected in a 95% for a filtration process with this support [62]. Moreover, it is also possible that metal inter-diffusion between support and Pd-based selective layer takes place after operating the membrane at high temperatures for long times. This phenomenon causes a marked decrease in the permeation capacity [89]. To overcome both drawbacks, the original support has to be modified prior to the incorporation of the H_2_ selective layer, as we detail in following sections [89,93,99].

On the other hand, ceramic supports provide a smoother surface with accurate control on porosity and narrow pore sizes distributions up to a few nanometres [38]. These properties facilitate the deposition of defect-free palladium layers with really low thickness and many researchers choose to use them as support for membranes [41,53,84]. Among some possibilities, the use of alumina, Al_2_O_3_ [102,103], is predominant, usually combining both α-Al_2_O_3_ and γ-Al_2_O_3_ particles in order to prepare asymmetric supports with big pores in the core to ensure greater permeabilities and smaller ones on the top layer to facilitate the palladium incorporation [71]. However, this material presents a thermal expansion coefficient noticeable different to that of palladium, besides a weak mechanical resistance, jeopardizing the integrity of the supported membrane, which is quite important on membrane reactors [91]. Other alternative less frequent is the use of yttria-stabilized zirconia (ZrO_2_-YSZ), with closer thermal expansion coefficient to that of palladium (10.0 × 10^−6^ °C^−1^), to prepare ceramic supports [85,96]. Anyway, different metallic or ceramic supports can be used to prepare totally dense supported Pd-based membranes, although a prevalent solution is still not reached. The advantages provided for ceramic ones are problems when using metal supports and *vice versa*, so different trends can be observed in literature. Some authors lean towards ceramic supports, mainly formed by alumina, in order to ensure the incorporation of an ultrathin Pd-based layer without defects, focusing on the membrane preparation, while other ones prefer to use the metallic supports thinking on real application of membranes in stainless-steel industrial devices.

Independently of constituent material of supports, the geometry is also important and quite a few configurations can be found in the literature, distinguishing mainly planar [100,101], tubular [71,78] and hollow fibre geometries [104,105]. In general, tubular geometries of both ceramics and metallic materials are prevalent in case of considering the use in a membrane reactor, while porous metals with planar geometry are most frequent in case of studying the membrane preparation with only purification purposes [106,107,108]. However, this situation has changed in the last years with the appearing of plate-type geometries and hollow fibres in design of trending reactor systems with micro-channels [109].

Table 1 summarizes the most frequent inorganic supports present in the literature for Pd-based membrane preparation, indicating important parameters such as material, geometry, average porosity and illustrative pore sizes. Several relevant manufacturers around the world have been considered, such as Mott Metallurgical Corp. (USA), Pall Corp. (USA), GKN Sinter Metal (UK), Inopor GmbH (Germany), TAMI industries (France) or NGK Insulators Ltd. (Japan). Currently, lower prices can be achieved for ceramic supports, despite they present smaller pore sizes than metallic ones, although their reutilization is not easy due to the frequent breaking during operation.

As previously mentioned, it is not common the direct use of commercial supports to prepare supported membranes, especially in case of metallic substrates. On the opposite, it is usual to carry out some pre-treatments and surface modifications of the support to improve the final quality of the membrane. In addition to conventional initial cleaning procedures, most of these modifications are focused on the improvement of layers adherence and/or the reduction of average pore sizes and roughness in the support surface to achieve thinner hydrogen selective layers. These treatments can be classified in three general categories: (i) chemical treatment, (ii) physical treatment and (iii) incorporation of an intermediate layer. Considering the great importance of these steps on the final properties of the membrane and its costs, some of the most relevant advances and extended practices are summarized in the next paragraphs. We have focused on metallic supports since, as mentioned above, they are the most suitable to use in membrane reactors to hydrogen production, which is the aim of the review. Moreover, it should be noted that the external surface of ceramic supports is not usually modified prior to deposit the selective layer due to the good original properties in terms of average pore diameter and surface roughness.

### 2.1. Chemical Treatment

The use of chemicals to modify the surface of supports is known as etching. It is commonly applied for polymers but it can also be used to modify some original properties of inorganic materials. These treatments consist of dipping the support in a corrosive solution, traditionally a strong acid and maintaining it at controlled temperature for a short period of time. The main effect of these treatments is to dissolve oxides thin films formed on the top of the supports, being also possible to remove part of the support bulk material. This action is primary determined by support composition, acid concentration, temperature and time of the treatment. Mardilovich et al. [110] used a solution of hydrochloric acid to treat a commercial stainless-steel support, achieving a noticeably increase of roughness on the surface of stainless-steel particles that form the support after only 5 min. of immersion. Moreover, the new treated surface evidenced better properties for the subsequent palladium incorporation as a pre-activated surface, increasing the plating rate and improving the adherence. A similar treatment was reported by Li et al. [111] but mixing the hydrochloric acid with some amount of nitric acid and Kim et al. [101] for preparing a supported Pd membrane over a porous nickel support. In this way, the etching pre-treatment of an inorganic support can provide benefits for the pre-activation of support surface and adherence of the selective layer at a relatively low cost, independently of using any other additional treatment such as mechanical modification or the incorporation of an additional layer.

### 2.2. Mechanical Treatment

A different alternative to modify original supports, mainly the metallic ones, can be carried out by the polishing of the external surface. The plasticity of the metal particles that form the support is used to reduce both external pore size and roughness through a mechanical treatment with an abrasive material. One of the first references about the use of this alternative to prepare Pd supported membranes, published by Jayaraman et al. in the nineties, utilized commercial sandpapers with different grit numbers to smooth the original surface of the support [112]. Particularly, they used commercial sandpapers with grades #320, #500 and #800. Later, Mardilovich et al. [110] used a similar polishing process with commercial sandpapers to modify the surface of porous stainless-steel supports. They indicated that it was possible to reduce both external average pore size and roughness, although most porosity was lost, decreasing the permeation capacity of the modified support up to 20% of the untreated one. Most recently, a similar technique based on the use of an abrasive sandpaper has been reported in the literature, as evidence the works published by Li et al. [111], Ryi et al. [113] or Pinacci et al. [114]. This polishing technique has not been only proposed for modifying the surface properties of supports, being also possible the reparation of defects in palladium thin films of supported membranes by mechanical treatments [115]. Despite this type of mechanical treatment is the predominant one, it is also possible to find some work in which high velocity shot peening with ion particles is used to achieve the plastic deformation of the metal particles of the support. However, the high cost of this alternative makes the traditional abrasive ones prevalent [116].

However, some researchers have critical opinions about these mechanical treatments due to the reduction of the permeation capability and adhesion properties of the thin selective layer, which in turn constrain the performance of the supported membrane. In this context, it is accepted that the adhesion between a support and a thin selective layer depends on the mechanical binding and anchoring effects. Consequently, it is necessary a minimal support roughness for ensuring a good adhesion of the top coatings [117,118]. This is clearly indicated in works published by Collins [119] and Huang [120], where larger pores and a certain external roughness in supports improve the adhesion of the thin coating layer. In this manner, it can be stated that it is necessary to achieve a compromise solution between the original surface modification and maintaining certain anchoring points to guarantee a suitable adherence of the Pd selective layer.

### 2.3. Incorporation of Intermediate Layers

In spite of using chemical and/or mechanical treatments, the incorporation of an intermediate layer between the commercial support and the top selective layer is the most preferred alternative to improve the external surface of the support. This option can be used simultaneously for different objectives such as modification of the original morphology, metal inter-diffusion mitigation, adhesion improvement of the Pd selective layer, corrosion prevention of support or even incorporation of first metal nuclei as pre-activated surface. The last one is usually the main reason to incorporate intermediate layers on ceramic supports due to these materials usually present a very smooth surface with very narrow pore sizes distribution, up to 3 nm [71,121] without need of additional modifications. However, metallic supports display a typical rough surface and wide pore sizes [62,71], being the incorporation of an intermediate layer a critical issue to achieve a really thin palladium layer. Considering the final target of the intermediate layer, its composition and thickness need to be adjusted at a reasonable cost and, up to now, a unique solution is not reached. 

**Figure 3:**
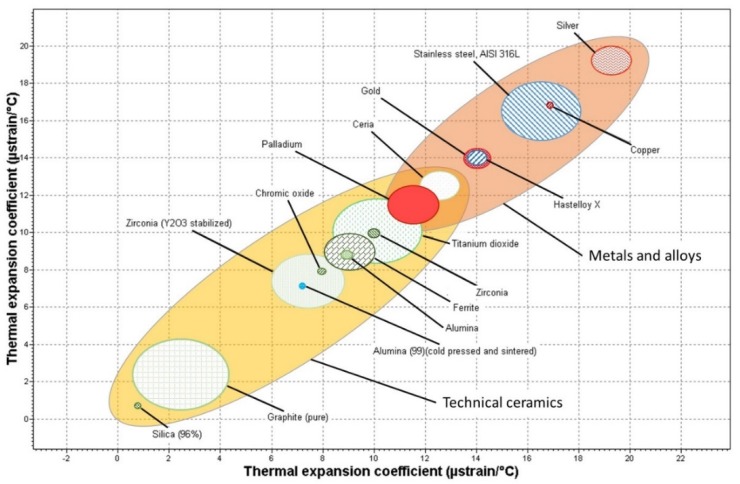
Thermal expansion coefficients for typical constituents of supported membranes for hydrogen separation.

Anyway, one of the most important factor to be considered is the compatibility between the different components of the supported membrane. Figure 3 shows the thermal expansion coefficient for some of the materials most frequently used as intermediate layer, besides common metal support raw materials (316L stainless-steel or Hastelloy X) and selective layer constituents (mainly palladium, silver, copper and gold). 

At large, small differences between the thermal expansion coefficients of the supported membrane elements are recommended to ensure enough mechanical resistance at operating conditions, usually at moderate or high temperatures. According to the data shown in Figure 3, cerium oxide appears as a very attractive alternative, with a thermal expansion coefficient between of palladium and common metallic supports (i.e., AISI 316L stainless-steel or Hastelloy X). This material was employed by Tong et al. [122] to modify a macro porous stainless-steel tubular support to prepare a supported Pd membrane with a selective layer of around 13 μm thickness. They evidenced a really good stability of the supported system after long-term experiments, obtaining almost equal hydrogen permeability to the theoretical value for a pure Pd membrane. A similar intermediate layer of CeO_2_ was prepared by Qiao et al. [123] to prevent intermetallic diffusion between a PSS support and a PdCu alloy selective layer. This intermediate layer was prepared through a sol-gel method and the modification of the original support also improved the adherence between the metallic support and the selective layer. 

In addition to CeO_2_, many other materials have been also successfully incorporated as an effective intermediate layer, even though they present different thermal expansion coefficient to that of the selective layer or the support. A first relevant group is formed by zirconium oxide and related materials. Some authors, such as Wang et al. [124] or Gao et al. [125], modify commercial PSS supports by the incorporation of ZrO_2_ particles to reduce the thickness of the hydrogen selective layer up to around 10 μm. A similar thickness was achieved by Tarditi et al. [93] by the incorporation of the ZrO_2_ particles through a vacuum-assisted method, while Lee at al. [126] reduced this thickness up to 3.5 μm for a better permeability. Other researchers added small amounts of yttria to the based-zirconia material in order to increase the structure stability of the material, obtaining an yttria-stabilized zirconia (YSZ) [50,117]. References in literature present the use of YSZ as an effective intermediate layer with the double aim of reducing the palladium thickness and preventing the intermetallic diffusion between support and selective layer, indicating sol-gel methods or atmospheric plasma spraying as successful techniques for the material incorporation [70,78,127]. 

Considering the relatively good surface properties of alumina as support, the use of this material as intermediate layer to modify the metallic supports has been also proposed by different authors. In this way, Yepes et al. [128] and Li et al. [129] decreased the original pore size of the metallic support by incorporating an alumina top layer that prevents possible inter-diffusion processes between the original support and the selective layer. Broglia et al. [130] reported the incorporation process of γ-Al_2_O_3_ particles by dip-coating onto a PSS support to achieve a totally defect-free Pd layer of around 11 μm. Chi et al. [131] detailed the use of different graded alumina particles for a better modification of commercial PSS tubes. They used particles with a size close to 10 μm to fill the widest pores and smaller particles (size around 1 μm) for a final smooth of the surface. Thus, they eventually achieved a thin free-defects Pd layer with less than 5 μm in thick and good thermal stability. Lee et al. [126] compared the effect of using Al_2_O_3_ and ZrO_2_ technical ceramics with similar thickness as support modifiers and they indicated that both materials act effectively as diffusion barrier, although the use alumina yield a lower membrane permeability.

Other conventional material used as intermediate layer is the SiO_2_, being possible to accomplish different functions such as surface support modifier, intermetallic diffusion limiter, perm-selectivity booster or even catalyst for some chemical processes. For instance, Nam et al. [132] modify a commercial 316L stainless-steel substrate by the incorporation of amorphous silica. In this way, they reduced the selective layer, constituted by a PdCu alloy, up to 2 μm but maintaining an excellent separation behaviour with hydrogen permeance of 8.37 × 10^−7^ mol·m^−2^·s^−1^·Pa^−1^ and H_2_/N_2_ selectivity of around 70,000 at 450 °C. Calles et al. [62] published the use of three different siliceous materials as intermediate layer for preparing supported Pd-PSS membranes: amorphous disordered silica, amorphous ordered silica (HMS) and crystalline silica (silicalite-1). In all cases, both roughness and pore size of the original supports were reduced and, consequently, the minimum Pd thickness required to obtain a defect-free selective membrane. The best results were obtained for the silicalite-1 material, reducing the Pd thickness up to 5 μm and yielding a hydrogen permeance of 1.423 × 10^−4^ mol·m^−2^·s^−1^·Pa^−0.5^ with a complete hydrogen selectivity at 400 °C. Similar modifications of metallic supports with microporous silica layers can be found for increasing the H_2_ perm-selectivity of the composite without any other additional layer [133] or even combined with palladium in a mixed-matrix structures [134]. Recently, these materials have been also applied on the top of finished supported Pd membranes in order to repair small defects and pinholes, significantly increasing the H_2_ selectivity with a very low cost [135].

Materials that combine silica and alumina are the well-known zeolites, crystalline materials with controlled pore sizes distribution and additional catalytic properties. Among the wide variety of possible structures, the use of zeolites NaA [136], NaX [137], Z-21 [138], FAU-type [139] and TS-1 [140,141] as effective intermediate or protective layers in membrane preparation can be found in the literature. On the whole, the higher cost of these materials limits their use to very specific processes, mainly for membrane reactors in which undesirable products are presented and the zeolite plays the role of both support modifier and catalyst. 

Other method quite simple to modify the support, with high reproducibility and reasonable cost, is the direct oxidation of 316L PSS supports in air atmosphere at high temperatures. This process yields a top coating of mixed Fe_2_O_3_-Cr_2_O_3_, which is able to prevent the inter-diffusion process [78]. Ma et al. [142] patented a controlled in-situ oxidation method to prepare Pd composite membranes over porous stainless-steel supports and thus, they achieve effective inter-diffusion barriers with thermal treatments upper than 600 °C. Following this pioneering work, other researchers such as Guazzone et al. [143] or Mateos-Pedrero et al. [144] modified PSS supports by the incorporation of metal oxides derived from an oxidation process at temperatures higher than 400 °C. Mostly, only slight modifications on the support surface can be observed after the thermal treatments due to the very limited thickness of the new oxide layer and, consequently, the Pd thickness is not reduced as much as when other alternatives are used. In case of using really high temperatures for the treatment (>700 °C), more oxides are generated, although in that cases the original porosity of the support drastically drops.

In the last years, some other materials have been investigated to develop more efficient intermediate layers and achieve better supported membranes. Some of these new materials are thin TiN thin layers obtained by sputtering [145], a combination of silver as diffusion barrier and aluminium hydroxide gel for filling in the biggest pores of the support [146], bi-metal multi-layers formed by staked layers of Pd and Ag [147], nickel [148] or even tungsten powders [47]. However, despite these promising results, a definitive solution has not yet been found. 

**Figure 4:**
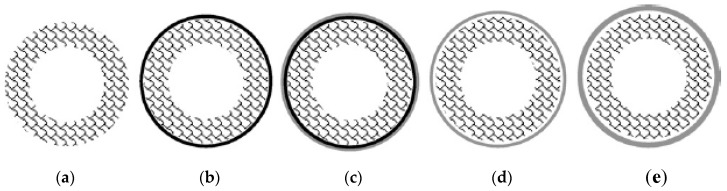
Use of a temporary intermediate layer for the preparation of a Pd-composite membrane: (**a**) original support; (**b**) polymer + support; (**c**) Pd layer + polymer + support; (**d**) Pd layer + small gap + support; and (**e**) defect-free Pd layer + small gap + support [150], with permission from © Elsevier.

One original alternative consists of using a temporary material to make the incorporation of the selective Pd layer easier. For instance, Tong et al. reported this methodology for the first time, employing an aluminium hydroxide gel or a polymer to modify the top surface of a PSS support. Then, they deposited the Pd layer over the modified surface and, finally, the temporary intermediate layer was removed in order to recover the original pores of the support [149,150]. Figure 4 collects the main steps carried out during this attractive method. Following this procedure, the authors prepare membranes with around 5 μm of palladium thickness that exhibited a maximum hydrogen permeation flux of 0.82 mol·m^−2^·s^−1^ with infinite hydrogen selectivity at 600 °C and ΔP 200 kPa.

Finally, despite the presence of an intermediate layer in ceramic substrates is less common, some examples can also be found in the literature. For instance, the work published by Hu et al. [151], in which a low-cost macroporous Al_2_O_3_ support is modified with graphite and clay from a conventional 2B-pencil. With this method, they achieved a totally defect-free supported membrane with a palladium thickness of 5 μm. In spite of the incorporation of intermediate layers onto the ceramic supports prior to incorporate the final selective coating is scarce, is possible to found some works that use this alternative to improve the surface activation, as published by Zhao et al. [152]. They used a Pd(II)-modified boehmite sol for modifying the original surface and achieved a thickness of the selective layer of only 1 μm. A very particular application of this methodology is the synthesis of pore-filled membranes, in which YSZ particles are used to modify the original surface of ceramic supports in a double layer. The aim is to get a good adhesion and uniform coating of the membrane film onto the support, as well as create a barrier that plays as protection of the Pd-selective layer [50]. More details about this alternative can be found in the next section, talking about recent developments for improving the metal deposition processes via Electroless Plating.

**Figure 5:**
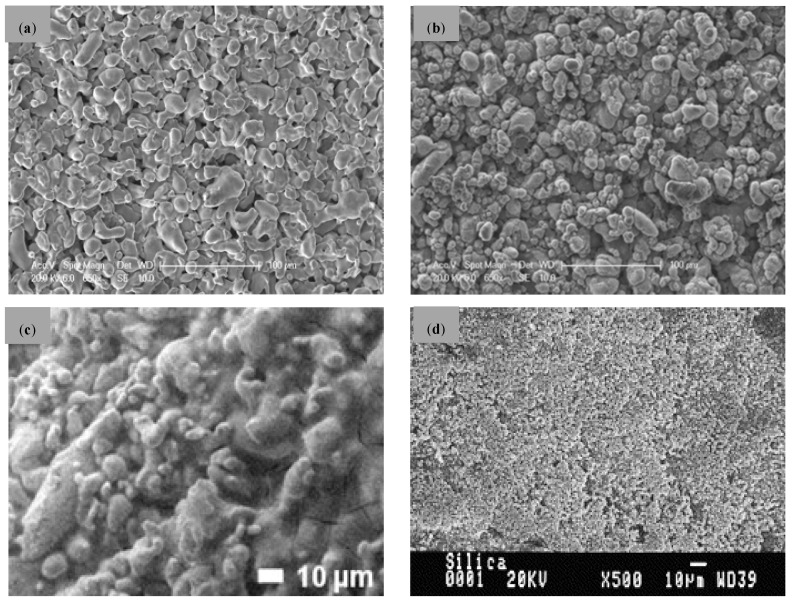

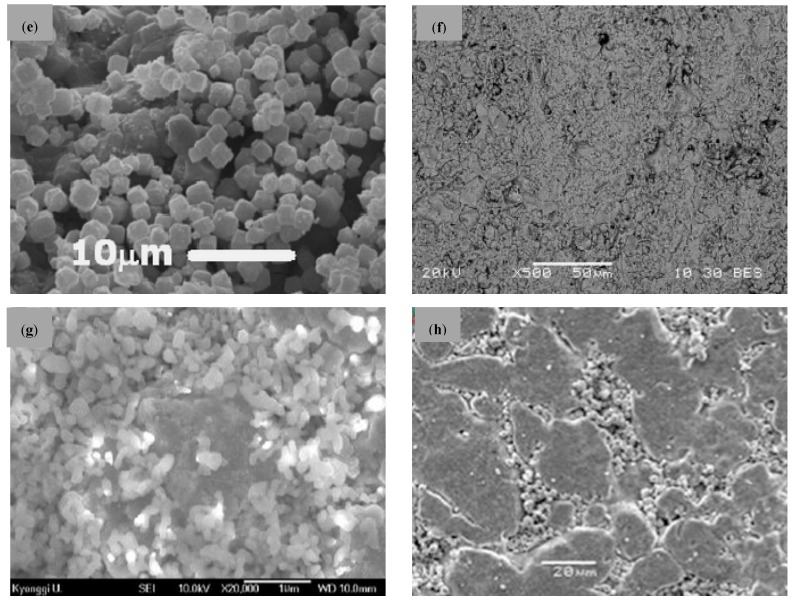
Porous stainless-steel supports before (**a**); and after the incorporation of different materials as intermediate layer: mixed oxides by calcination in air (**b**); alumina (**c**); amorphous silica (**d**); zeolite (**e**); zirconia (**f**); ceria (**g**); and tungsten (**h**). Figure adapted from originals published in [47,51,78,99,132,136,153], with permission from © Elsevier.

The morphology of the external surface of a typical commercial metal support and its modification after the incorporation of some of the previously described materials as intermediate layer are collected in Figure 5. As it can be seen, the original PSS surface is practically covered after the incorporation of the different materials, obtaining a very homogeneous external surface while surface roughness and original pore sizes are significantly decreased. 

The most relevant information about the wide alternatives included in this section to modify commercial raw supports has been also summarized in Table 2. Support nature and modification alternatives are collected, as well as other relevant parameters such as composition and thickness of H_2_ selective layer and permeation properties of the final supported membrane.

## 3. Palladium Incorporation by Electroless Plating

### 3.1. Electroless Plating Standard Method

The term Electroless Plating (ELP) was coined for the first time in the middle forties by Brenner and Riddell to define the metal deposition in the absence of an external source of electric current [154]. The application of ELP technology to the palladium incorporation on porous supports has been widely used to prepare hydrogen selective membranes for years. As it is previously mentioned in the introduction, this technique does not require any expensive equipment and neither high operational costs due to the absence of electrodes and external electricity sources. Moreover ELP is able to create homogeneous films on complex geometries and non-conducting materials [111,155,156,157], being the option usually preferred over other methods. Here, a general description of the method is presented, including the most relevant advances carried out during the last years in case of using both metallic and ceramic supports. 

Essentially, the use of ELP for the preparation of H_2_-selective membranes is based on the palladium deposition (or related alloying materials, as it is discussed later) onto a support target surface from an aqueous solution containing the metal precursor. Usually, this precursor is dissolved and stabilized with some ligand in order to form a complex prior to be reduced through a controlled autocatalytic chemical reaction [158,159]. In recent years, most published manuscripts use ammonium hydroxide and ethylenediaminetetraacetic acid to complex the palladium precursor. On the other hand, hydrazine is preferred as reducing agent due to the generation of nitrogen as unique by-product of the chemical reaction, avoiding other prohibited deposits in the film, i.e., phosphorous [157,159,160]. Hydrazine is a powerful reducing agent in both acid and alkaline media. The reduction of higher valent metal ions to lower metal ones or to the zero valent state is possible depending on the reaction conditions [161,162,163]. Hereunder, the main chemical reactions involved in the process for palladium deposition are:

(2) $$\begin{array}{c} 2\mathrm{Pd}{\left({\mathrm{NH}}_{3}\right)}_{4}^{2+}+4e^{-}\to 2{\mathrm{Pd}}^{0}+8{\mathrm{NH}}_{3}{\;E}^{0}=0.95\;V\\ N_{2}H_{4}+4{\mathrm{OH}}^{-}\to N_{2}+4H_{2}O+4e^{-}{\;E}^{0}=1.12\;V \end{array}$$

being the global reaction of the process as follows:

(3) $$2\mathrm{Pd}{\left({\mathrm{NH}}_{3}\right)}_{4}^{2+}+N_{2}H_{4}+4{\mathrm{OH}}^{-}\to 2{\mathrm{Pd}}^{0}+8{\mathrm{NH}}_{3}+{\;N}_{2}+4H_{2}{O\;E}^{0}=2.07\;V$$

In order to achieve a homogeneous Pd deposition, good adherence and reasonable induction times to spontaneously initiate the chemical reactions, the supports need to be seeded with a first nano-sized Pd nuclei before the main plating step [164]. Conventionally, this step has been carried out by repetitive immersions in acidic tin and palladium solutions, also known as sensitization-activation treatment [165]. However, some studies advise problems in membrane stability at high operating temperatures caused by tin residues, which lead to the formation of defects and pinholes in the Pd film, as Paglieri et al. indicated for the first time in the late nineties [166] and other authors endorsed most recently [167]. A detailed study about the correlation between presence of tin residues and membrane stability has been lately published by Wei et al. [167]. Considering these negative effects of classical sensitization-activation treatments, alternative methods avoiding the use of tin solutions have been proposed. Different approaches have been used, such as, the use of activated particles with Pd nuclei for intermediate layers preparation [125,127,146,168,169]; catalysed anodic alumina surfaces to facilitate the Pd electroless plating [170]; the increase of nuclei deposition rate and rupture of conglomerates by applying ultrasounds [171]; the incorporation, decomposition and reduction of a palladium acetate solution in chloroform onto the surface [172]; or, directly, the generation of nano-sized Pd particles by direct reduction of a highly diluted solution with a mixture of ammonia-hydrazine [51,71]. However, up to date it has not been found a better solution and the classical method remains as the top choice for many researchers [28,46,161].

### 3.2. Recent Developments in Electroless Plating

In last years, great efforts have been carried out to reduce the overall cost of Pd-based membranes manufacturing, mainly focusing in reducing the palladium layer thickness but ensuring absence of defects in the coating [35,37,38,173]. As previously commented, one strategy is based on the preparation of supported membranes, which usually involves the modification of raw supports to facilitate the incorporation of an ultrathin free-defects palladium layer [21,62]. Other strategies are focused to improve the metal deposition process, particularly the electroless plating, to achieve better adherence, homogeneity, greater pores coverage or, in a general way, better stability of the Pd-based H_2_-selective layer with minimum thickness. Along following lines, the most relevant developments in this context are presented, particularly focusing on the advances published in recent years. 

Thereby, Uemiya et al. [174] increased the metal incorporation rate by immersing the porous support in a solution containing hydrazine prior to each electroless plating step. Other authors tried to improve the palladium incorporation in deep areas on the surface, where deposited metal particles effectively close the pore mouths of the support and complete a fully dense and continuous layer by the bridge mechanism [175]. Zhao et al. [152] and Zhang et al. [176] reported the use of vacuum in the inner size of the supports to achieve uniform microstructure of Pd layer with a lower average thickness to that of conventional electroless plating. Similar results were obtained by other researchers, such as Yeung [177], Souleimanova [178] or Li [179] when osmotic effect is generated between the plating solution and an aqueous sucrose solution. 

**Figure 6:**
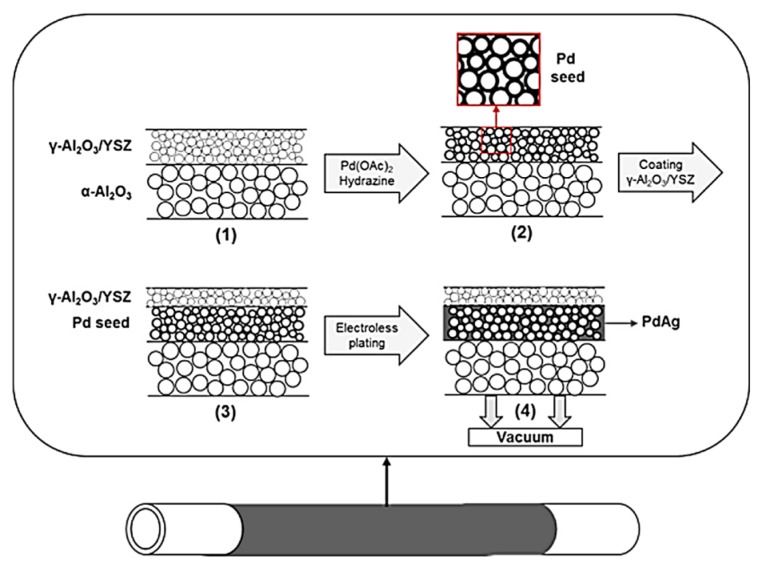
Procedure to prepare pore-filled type membranes [90] with permission from © Elsevier.: (1) Incorporation of a first γ-Al_2_O_3_/YSZ layer; (2) Pd seed on smaller ceramic particles; (3) incorporation of a top additional γ-Al_2_O_3_/YSZ layer and (4) incorporation of a Pd-based layer by vacuum-assisted ELP.

Pacheco Tanaka et al. [50,52,180] went a step further by preparing supported membranes in which the incorporation of Pd or Pd-based alloys was carried out by vacuum-assisted electroless plating between two zirconia oxide ceramic layers, one of them activated with a previous Pd seed, deposited onto a tubular alumina support. This particular kind of membranes in which the selective layer is placed into a sandwich-type structure was denoted as pore-filled type membranes. The main advantages outlined by the authors includes the ability to operate the membrane below the critical temperature and to maintain a fully mechanical stability, unlike other supported membranes based on a conventional external coating, where fatal damages usually occur. Moreover, the sandwich structure also provides to the selective layer an additional protection against poisoning. Recently, a clear scheme describing in detail the fundamentals of this alternative has been reported by Arratibel et al. [90], as shown in Figure 6 (with permission).

On the other hand, different studies are focused on modifying the plating baths composition to improve the final properties of the palladium film. In this context, it has been demonstrated that conventional electroless plating baths containing ethylenediaminetetraacetic acid (EDTA) present good stability at different temperatures, although it results in limited purity of the palladium layer due to the incorporation of carbon deposits from the EDTA complex within the metal particles [97,181,182]. These carbon deposits could diminish the membrane performance by CO_2_ formation at some operating conditions. Thus preparation of free-EDTA baths has been also investigated, achieving acceptable palladium deposition yields with good stability of plating baths in absence of these stabilizer [97,181,182].

Other authors have studied the influence of fluid dynamics between support and plating bath. Thus, the rotation of the support during the electroless plating has been found to increase the plating rate and the homogeneity of Pd layer, as reported Chi et al. [183]. Compared with static ELP, the use of rotation of the support during the process reached more uniform and smoother surfaces of Pd membranes, which in turns enhances the stability of the supported system. These authors reported a membrane permeance of 3.0 × 10^−3^ mol·m^−2^·s^−1^·Pa^−0.5^ with ideal hydrogen separation factor upper than 400 for only 5 μm thick (T = 400 °C, P = 4 bar).

Despite the effort in the research to improve both quality and cost-efficiency of Pd membranes, other many studies are focused on diminishing the rejected membranes due to the presence of defects or cracking during the fabrication processes. In this mean, novel alternatives to repair possible defects generated on the Pd surface have been recently reported. Thus, Li et al. [179] use the fundamentals of previously reported osmotic effect to incorporate Pd particles preferentially in defect areas for repairing the Pd layer. Following this procedure, they ensure the complete disappearance of defects and a consequent meaningful increase in the ideal hydrogen separation factor without noticeable reduction of the permeation flux nor thickness growth. With similar fundamentals, point plating has been also proposed by Zeng et al. [184] to repair located defects in supported Pd-based membranes. In this case, the method forces the chemical reaction for palladium reduction around defects by feeding both metal source and hydrazine baths from opposite sides of the supported membrane.

**Figure 7:**
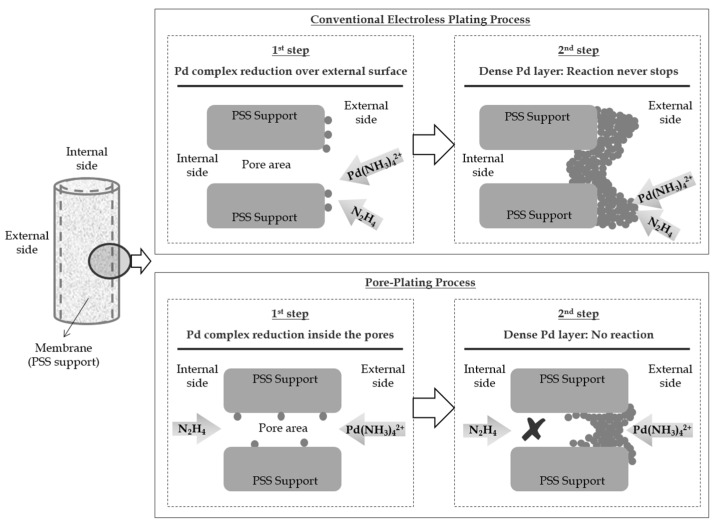
Pd incorporation around pores in both conventional electroless plating (ELP) and pore-plating (ELP-PP) alternatives [185], with permission from © Elsevier.

Based on these repairing procedures, other researchers have recently reported the separated supply of Pd source and reducing agent bath to prepare Pd-based membranes directly on rough commercial PSS supports [49,51,71,78,79,80]. This novel procedure, denoted as Electroless Pore-Plating (ELP-PP), uses the wall of the support itself to maintain separated both Pd source and hydrazine solutions. At these conditions, hydrazine preferentially diffuses through the pores of the support and reacts with the amino-palladium complex near the pore area. Ideally, in case of proper activation of the inner pore surface, this reduction initiates from the internal porosity of the support in a similar way to the sealing method previously [49,51,185] asserted that it is possible to save palladium source and to minimize the number of rejected membranes following this methodology, consequently reducing the overall cost of membrane preparation. This is possible because the contact between reactants turns progressively difficult during the Pd incorporation up to the complete block of pores, moment at which the process stops. The comparison of both conventional ELP and ELP-PP alternatives is shown in Figure 7. This method hinders the increasing of palladium incorporation after blocking the pores, in contrast to the behaviour reached by conventional ELP, resulting in a fully dense film with minimum thickness.

In spite of the preferential incorporation of Pd inside the pores of the support, authors revealed the generation of an external film on both commercial and modified PSS supports caused by the wide variety of pore diameters in these supports [49,51,185]. The hydrazine cannot pass through the smallest pores because they become fully closed by palladium particles in a relative short time, while the reducing agent can diffuse through the widest ones, partially covered, until the outer surface in contact with the palladium bath, where the external layer is formed. In this manner, it is obvious that several parameters affect the ELP-PP process: (i) pore characteristics of support (average pore diameter and porosity), (ii) reducing and metal plating baths formulation and (iii) ratio between membrane length and volume of solutions. In fact, supports with smaller pores, achieved by direct oxidation of commercial supports in air, provide membranes with an apparent thickness around 10 μm, a half of the value reached in case of using unmodified supports (20 μm). However, this apparent thickness, determined by gravimetric analysis, is 2–6 μm greater than the real value obtained from SEM characterization due to the Pd introduction in the pores of the support is not considered for the estimation. All membranes obtained by this ELP-PP alternative exhibited high stability at different simulated and real operating conditions in a WGS membrane reactor with permeances in the range 1–6·10^−4^ mol·m^−2^·s^−1^·Pa^−0.5^ and complete H_2_ selectivity [51]. Within the recent past, this novel method has been also reported for preparation of supported membranes on ceramic supports with noticeable smaller pores respect to typical PSS supports, confirming the great importance of support characteristics on the plating performance (primarily average pore diameter and pore size distribution). In this way, thinner membranes were achieved even though palladium is still present in both internal pores and external surface [71].

**Figure 8:**
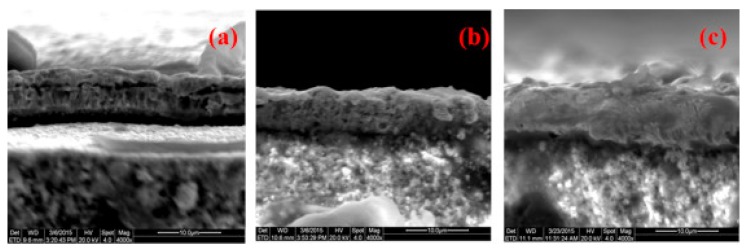
Microstructural modification on Pd films prepared by ELP after different thermal treatments: (**a**) as prepared; (**b**) 168 h at 550 °C; and (**c**) 72 h at 700 °C [42], with permission from © Elsevier.

Finally, some researchers propose the improvement of membranes properties, mainly the increase of permeation rate and thermal stability simultaneously to the presence of defects is decreased, by using a further thermal treatment step (>640 °C) after the palladium plating [42]. Although this alternative is not strictly an improvement of the metal deposition process, it can be used to enhance the previously prepared membrane. In fact, a heat treatment of as-prepared membranes improved the Pd layer microstructure, achieving a densification of the metal film, as it can be seen in Figure 8.

Like previous information about main alternatives for modification of commercial supports, here we summarize the most relevant advances for Pd incorporation by electroless plating in Table 3. Key improvements and experimental details are summarized beside information about support material, support modifications, thickness of selective layer and permeation properties.

## 4. Pd-Alloy Membranes

Independently of using conventional or improved electroless plating processes, many researchers endorse the preparation of alloys in which palladium is combined with some amounts of other metals in order to improve the permeation behaviour, the thermal and mechanical stability and the poison tolerance of the membrane [56,186,187,188]. Thus, in this section we present an overview of most frequent Pd-based alloys, detailing the preparation procedures and main reported benefits, as well as recent trends and future perspectives for new formulations with improving properties.

Pure Pd usually suffers the so-called hydrogen embrittlement phenomenon due to the lattice expansion provoked by the α to β phase transition that occurs when the metal is exposed to hydrogen atmosphere at temperatures and pressures below 298 °C and 2 MPa, respectively. This phase transition generates tensile stress, especially in case of tubular geometries, which often leads to cracking the Pd layer and thus, a subsequent loos of hydrogen selectivity of the membrane. This drawback can be avoided by working at operating conditions above the mentioned critical point when membrane is exposed to hydrogen or modifying the Pd phase diagram [189]. The last option can be realized by alloying pure Pd with other metals, i.e., silver [52,106,190,191,192], copper [99,102], ruthenium [187,193] or gold [194,195]. It is demonstrated that Pd-based alloys with specific concentrations of these metals modify the metal-hydride phase diagram avoiding the mentioned embrittlement phenomena [28]. 

Other problem that negatively affects to the permeability of dense Pd-based membranes is the irreversible poisoning by chemical contaminants, such as carbon monoxide or sulphur. These molecules are chemisorbed over the metallic layer, being also possible a chemical reaction with hydrogen to form species that block the active sites on the surface and hinder the hydrogen permeation. Some alloys help to avoid this poisoning effect while maintaining an ideal complete hydrogen separation factor [90], even in presence of sulphur compounds that traditionally causes irreversibly poisoning in pure Pd films [28,102,186,196]. 

### 4.1. Alloy Preparation

The preparation of efficient Pd-based alloys by electroless plating with an accurate composition is currently one of the most important milestones for industrial membranes implementation. Physical vapour deposition provides multiple possibilities for incorporating different metals to the membrane with a really good control of the alloy composition [61,197,198,199]. However, this technique has some difficulty to generate defect-free layers on rough surfaces and high investments costs [60]. Thus, at the present date, the metal incorporation by electroless plating is widely adopted [182]. 

**Figure 9:**
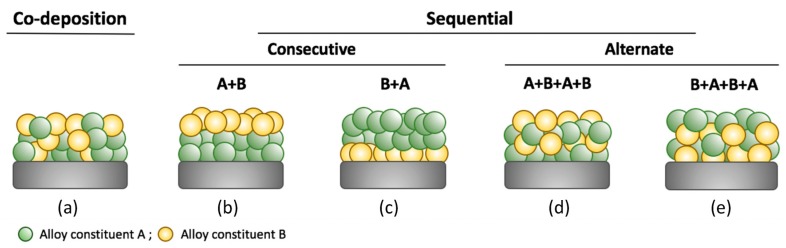
Different possibilities to prepare binary alloys by electroless plating: (**a**) co-deposition; (**b,c**) sequential deposition; (**d**,**e**) alternative deposition.

In general, the incorporation of metals by electroless plating for preparation of alloys can be carried out in different ways after a previous activation of the support, as illustrated in Figure 9. First, a unique plating bath containing all alloy constituents, i.e., materials A and B, can be used to deposit simultaneously all of them, being denoted as co-deposition (Figure 9a). In this case, the alloy constituents are randomly distributed in the selective layer with similar composition in both longitudinal and transversal directions. Thus, the following thermal treatment to form the alloy is favoured. However, this option is only possible in case of using metals with analogous properties that can be reduced in similar conditions, i.e., palladium and silver with comparable bath compositions and identical reducing agents. However, kinetics of the reduction process can be different for each component and, consequently, it is not easy to define the bath conditions to achieve a desired alloy composition [28,74,200,201]. 

Other possibility to prepare Pd-alloy membranes is based on sequential depositions of each constituent, incorporating all required amount of material B onto a previous layer formed by the material A (Figure 9b) or *vice versa* (Figure 9c), denoting both alternatives as consecutive methods. The alternation of different layers formed by each constituent until achieving the desired composition and layer thickness is also viable (Figure 9d,e, alternate methods). In these cases, it is possible to incorporate metals from different plating baths by using the same or different reducing agents on the condition that galvanic displacement does not occur. The kinetics of deposition processes can be easily controlled and the final alloy composition is determined by recurrences of each constituent plating. The most relevant drawback of sequential depositions is the difficulty to achieve a good alloy homogeneity through the whole layer thickness [202]. 

Anyway, a further thermal treatment is always required to achieve the diffusion of atoms within the solid material to form the alloy, independently of using sequential or co-deposited alternatives for the incorporation of metals. This process, also known as annealing, can be carried out under inert atmosphere (usually by using Ar, He or N_2_ as inert gas) [99,146,203] or in presence of hydrogen [52,94,187,200,204]. Traditional annealing processes for Pd-based membranes use inert environment at ambient pressure and require quite long times [53]. However, recent developments prefer faster processes in pressurized hydrogen atmosphere. In this case, it is proposed that dissolved hydrogen forms vacancies in the crystal lattice of palladium favouring the mobility of other alloy constituents and, consequently, reducing the time required to obtain the alloy [195]. As previously mentioned, layers prepared by co-deposition need softer thermal treatments (shorter times or lower temperatures) for annealing as compared with layers generated by sequential deposition (either consecutive or alternative) [95,202]. Taking into account that preparation of alloys with accurate control is a decisive challenge for the large-scale application of Pd-based membranes [205], following sections summarize the most relevant advances in this field, distinguishing the preparation of binary and ternary alloys.

### 4.2. Binary Alloys

**Figure 10:**
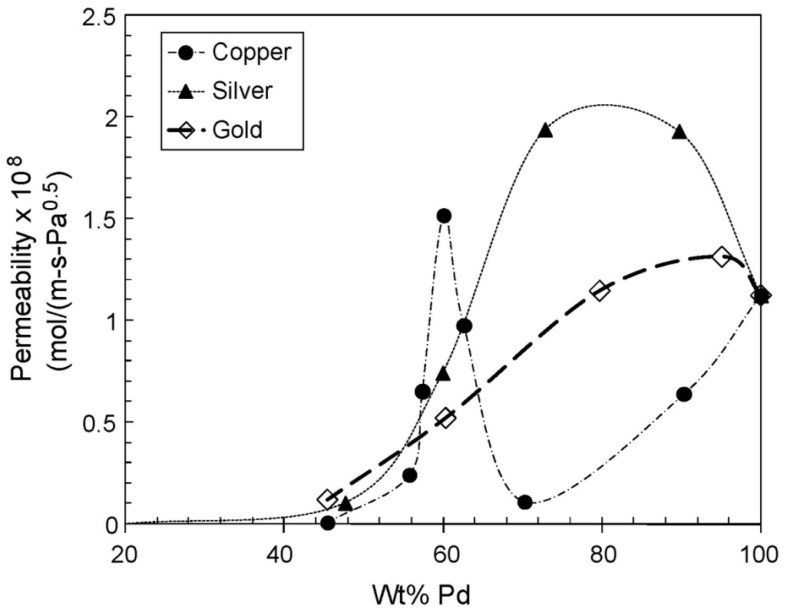
H_2_ permeability at 350 °C for different Pd-based alloys containing Ag, Cu and Au [206], with permission from © Elsevier.

Among the large number of feasible alloys from different metal pairs, the Pd-based binary alloys are the most frequently studied and used for hydrogen production. As mentioned before, alloying palladium with other component can avoid the hydrogen embrittlement as well as improve mechanical and chemical properties. In some specific cases, the hydrogen permeability may be even increased, depending on the alloy composition (Figure 10). Some alloys can improve the hydrogen permeability of the membrane only in a narrow composition window, while others also work in a wide range of compositions. Deviations from these target compositions or differences in composition inside the bulk metal may deteriorate noticeably the permeation behaviour respect pure palladium. For instance, this occurs when exceeding 36 wt % or 21 wt % in case of alloying with silver or gold, respectively. For palladium-copper alloys, small deviations from a target Pd_60_Cu_40_ value reach to a drastic decrease in hydrogen permeability.

#### 4.2.1. PdAg Membranes

One of the first alternatives to prepare binary Pd-based alloys is based on the addition of silver, used since the eighties for separation of hydrogen isotopes [207,208]. It is widely reported that mechanical strength against hydrogen embrittlement is significantly improved after silver incorporation on a palladium layer [201], as well as original hydrogen permeability can be also increased for some particular conditions [209]. As previously shown in Figure 10, the addition of silver on bulk palladium to prepare a binary PdAg alloy increases the membrane permeability in a wide range of compositions, from very low Ag percentages up to around 36 wt % [206]. Particularly, it is proved that using a Pd_75_Ag_25_ composition the hydrogen permeation reached a maximum and, thus, many researchers around the world have adopted this composition as main target for membrane preparation. Concerning the preparation procedure to obtain this alloy by electroless plating, both co-deposition [95] and sequential deposition [202] alternatives can be widely found in literature.

The research group headed by Yi Hua Ma has reported some studies on the binary PdAg alloy [83,210]. For example, one of the most interesting ones, published by Rajkumar et al. [211], presents the results obtained for incorporating Ag by both electroless and electro-plating techniques, after a first Pd incorporation by electroless plating. These PdAg supported membranes were prepared directly onto a commercial porous Inconel supports of 0.1 μm media grade, obtaining H_2_ selective layers under 10 μm thick with complete He retention up to a transmembrane pressure difference of 10^5^ Pa. All membranes were annealed during 24 h at 550 °C in H_2_ atmosphere. They observed that electro-plated Ag exhibited an optimal penetration in the pores of the support, although a non-uniform growth with dendritic morphology was achieved. In contrast, the use of electroless plating for silver incorporation provides a uniform growth without dendritic morphology and a lower penetration into the pores.

The contributions of the research group headed by Laura Cornaglia, based on the use of PdAg membranes prepared by electroless plating, are also widely reported in the literature [201,209,212,213,214]. One example is the work published by Bosko et al. [202], in which PdAg supported membranes were prepared by sequential electroless plating on tubular stainless-steel supports with thickness ranged from 20 to 26 μm. The supports were previously modified with both α-AI_2_0_3_ and γ-AI_2_0_3_ particles by a vacuum assisted-coating method. All membranes were annealed at 500 °C, exhibiting a hydrogen permeability of 3.1 × 10^−4^ mol·m^−2^·s^−1^·Pa^−0.5^ at 450 °C and 100 kPa and a H_2_/N_2_ ideal selectivity of around 954 at same conditions. They observed that annealing the membranes at higher temperatures created defects which deteriorated the selective layer, obtaining lower selectivity and higher permeability. 

**Figure 11:**
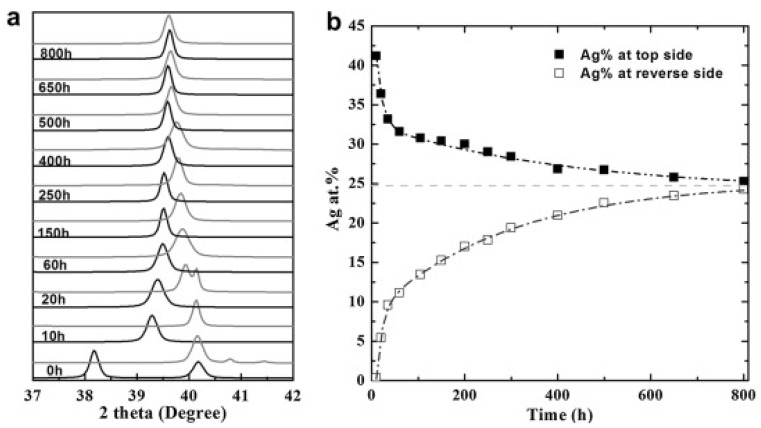
(**a**) 111 XRD reflections from the top (black) and reverse surface (grey) of a PdAg membrane during alloying at 550 °C and (**b**) convergence of the corresponding alloy lattice parameters [53]**,** with permission from © Elsevier.

Others works based on electroless-plated PdAg membranes to be highlighted are those of Andreas Goldbach and co-workers. Recently, Zeng et al. [53] reported the use of sequential electroless plating to prepare H_2_ selective PdAg membranes on Al_2_O_3_ tubular porous asymmetric supports. They incorporated the palladium prior to the required amount of silver to achieve a final composition of Pd_77_Ag_23_, after a thermal treatment at 500 °C in the presence of H_2_ between each deposition. Additionally, intermediate surface activations with Pd seeds were also carried out. In this way, membranes with a final thickness in the range 2.3–2.5 μm were achieved. Despite the limited thickness of these membranes, authors indicated the need to extend the annealing treatment up to 800 h maintaining a temperature of 500 °C under atmospheric H_2_ pressure. The progress of annealing between palladium and silver was monitored by XRD during the entire process as it is shown in Figure 11, to assess the formation of a homogeneous PdAg alloy. These membranes exhibited a H_2_/N_2_ selectivity between 3770 and 5600, with permeance values ranged from 5.77 to 3.86 × 10^−8^ mol·m^−1^·s^−1^·Pa^−0.5^, respectively. Similar to other researchers, they also reported the frequent membrane failure during the fabrication process due to the fragility of ceramic supports [89,95].

PdAg membranes prepared by Tecnalia innovation centre are also relevant. For instance, Ekain et al. [95] have recently reported a procedure to prepare thin PdAg membranes (thickness of around 3.2 μm) on tubular ceramic supports by using Pd and Ag simultaneous electroless plating during 210–240 min. The final alloy was achieved after annealing at 550 °C for 2 h, using N_2_ carrier gas for both heating and cooling rates but a mixture 10H_2_-90N_2_ when the annealing temperature has been reached. These membranes exhibit a H_2_ permeance of 3.10 × 10^−6^ mol·m^−2^·s^−1^·Pa and ideal selectivity in the range 8000–10,000, calculated for a H_2_ partial pressure of 1 bar and 400 °C. As other studies, these authors indicate that the use of ceramic supports generates fragile membranes, despite the good quality of the Pd-based selective layer. In this case, authors specifically recommended to avoid exceeding a maximum torque value when coupling the membranes into the reactor, usually made in stainless-steel. 

#### 4.2.2. PdCu Membranes

The use of copper as alloying element of Pd-based membranes not only improves mechanical strength against hydrogen embrittlement, also increases slightly the permeation rate as compared with palladium pure membranes while retaining the permeation capacity in gas mixtures containing sulphur compounds [205]. Moreover, copper is quite cheaper than palladium and thus, the percentage reduction of the last one in the selective layer (optimal composition around Pd_60_Cu_40_) reduces its cost. The preparation of PdCu membranes by electroless plating is usually carried out by sequential incorporation of palladium in first place and the copper, followed by an annealing treatment at high temperature. In this case, stable co-deposition is really difficult due to the different nature of each metal and the order of incorporation is also determined by the galvanic displacement of copper by palladium due to the lower reduction potential of the first one [215]. 

**Figure 12:**
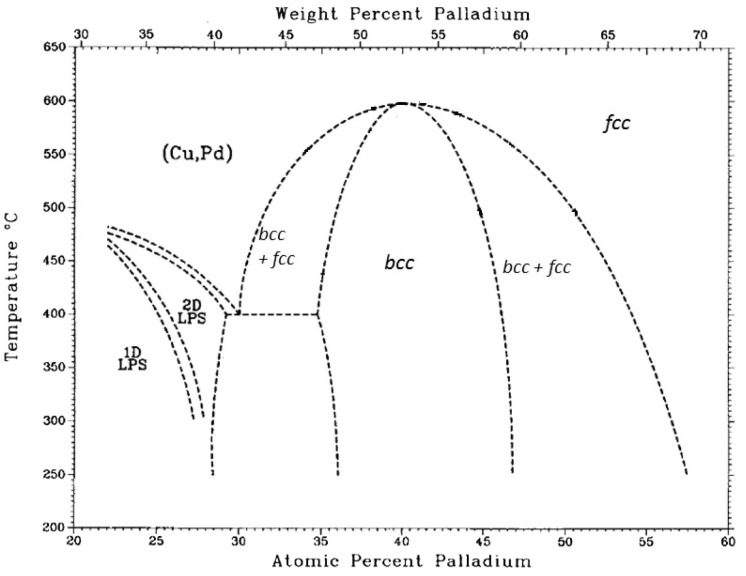
Pd-cu phase diagram [205], with permission from © Elsevier.

However, the use of PdCu alloys with face-centred-cubic metal structure (Figure 12) is restricted to a range of composition due to the drastic fall in H_2_ permeate when small variations in the 40% content of Cu are produced. 

Moreover, sulphur inhibition depended strongly on temperature. Zhao et al. [54] developed PdCu membranes on ceramic supports by using sequential deposition of metals via electroless plating and performed tests to analyse the influence of H_2_S. They revealed that single gas H_2_ permeation rates were significantly reduced after tests performed with 35 ppm H_2_S at 400 °C, although they fully restored the original operation capacity of the membrane after flowing H_2_ at 500 °C. However, the precedent flow rate obtained for N_2_ leaks increase noticeably (thus, decreasing α_H2/N2_ up to 1194), presumably due to sulphide formation in defect sites at lower temperatures.

The research group headed by Douglas J. Way maintains a wide activity on the study of PdCu systems for years [102,181,216,217]. Among all of them, it can be pointed out the preparation of PdCu alloys on tubular porous supports of symmetric α-alumina published by Roa et al. [84]. In this work, the incorporation of the H_2_ selective layer was carried out by sequential electroless plating of both metals, palladium and copper, followed by an annealing treatment at high temperature in flowing H_2_. Thus, a PdCu membrane with 11 μm thick was obtained, exhibiting a H_2_ permeate flux of 0.8 mol·m^−^^2^·s^−^^1^ at T = 450 °C and ΔP = 345 kPa with a H_2_/N_2_ ideal separation factor of 1150. 

Similar supported membranes based on electroless deposition of a Pd_60_Cu_40_ alloy layer were prepared by Qiao et al. [123] but using porous stainless-steel modified by the incorporation of a CeO_2_ as intermediate layer. In this work, copper was incorporated at room temperature on a first Pd layer prepared at T = 55 °C and a further annealing treatment at 480 °C under hydrogen atmosphere. The alloy layer thickness was around 8 μm, that yielded a hydrogen permeability of 5.92 × 10^12^ mol·m^−^^2^·s^−^^1^ at T = 450 °C (pressure was maintained at 10^5^ Pa). No nitrogen permeate was detected at ΔP = 10^5^ Pa and room temperature.

#### 4.2.3. PdAu Membranes

The addition of gold in a palladium layer brings out similar benefits to that of copper in Pd-based alloys, mainly attending to sulphur tolerance [91], although the cost of the resulting membranes is higher due to the higher cost of gold respect to that of copper and the need of a higher content in palladium for these types of alloys, usually upper than 80% [206]. However, the range of compositions in the PdAu that exhibits FCC structure without phase segregation, which ensures greater permeability than pure Pd membranes with diverse alloy formulation, is wider [205]. This fact is very beneficial in the membrane preparation since it is possible to achieve the alloy varying the gold content up to 21%, while maintaining improved permeation and sulphur tolerance. Among these possibilities, a composition target of Pd_90_Au_10_ seems to offer the best properties in the resulting membrane [206]. 

Attending to the preparation of these membranes, the combination of Pd electroless plating with a consecutive Au incorporation by galvanic displacement is the procedure widely described in most published works. Thus, Yi Hua Ma and co-workers prepared PdAu membranes that ensure good resistance to sulphur presence in a mixture 54.8 ppm H_2_S/H_2_ at temperatures ranged from 350 to 500 °C [94]. These membranes, prepared on a PSS support, have a gold content around 8–11 wt % with a total thickness below 15 μm. PdAu membrane experienced a decline in H_2_ permeance by ∼85% after the H_2_S exposure. Despite this, a value of ∼65% of the original permeance could be recovered in pure H_2_ at the poisoning temperature of 400 °C.

Similar PdAu alloys were also prepared by Tarditi et al. [93], in a ZrO_2_-modified porous stainless-steel support. Both metals, palladium and gold, were incorporated by sequential electroless deposition: palladium was first deposited at 50 °C in two steps, while a unique step at 60 °C was used for incorporating the gold. This process was repeated several times up to obtain a N_2_ non-permeable membrane at room temperature and ΔP = 10^5^ kPa at, obtaining a total thickness of around 10 μm. Finally, the membranes were annealed at 500 °C in H_2_ atmosphere to promote the alloy formation. A H_2_ permeation flux of 0.14 mol·s^−^^1^·m^−^^2^ was obtained at T = 400 °C and ΔP = 100 kPa, showing a H_2_/N_2_ ideal selectivity upper than 10,000. It was also possible to recover ∼65% H_2_ flux, in a Pd_91_Au_9_ membrane with an initial permeability of 1.1 × 10^−8^ mol·m^−1^·s^−1^·Pa^−0.5^ at 400 °C after 24 h exposure of 54.8 ppm H_2_S in H_2_ [201].

#### 4.2.4. Others Binary Alloys

The previously mentioned binary alloys based on palladium, silver, copper and gold are the most common alternatives used by researchers for H_2_ selective separation. However, it is also possible to find the combination of palladium with other metals to achieve additional advantages in cost-reduction or permeation behaviour. In this context, the principal limitation to explore new alloys it is the chance to incorporate the metals by electroless plating, being preferred to use the magnetron sputtering [61,199]. However, as we previously detailed, the use of electroless plating for H_2_ selective membranes preparation is recommended in terms of cost efficiency [33]. In this context, the use of nickel [148,218] or platinum [85,187] to prepare Pd-based alloys by electroless plating has been explored. Pd-Ni alloy supported membranes show long-term thermal stability at 300 °C under hydrogen permeation. An example of this alloy can be found in the work published Lu et al. in which Pd and Ni were consecutively incorporated by ELP on a capillary α-Al_2_O_3_ substrate [218]. The PdNi selective layer thickness is ∼7 μm and shows a hydrogen permeance of 2.74 × 10^−3^ mol·s^−1^·m^−2^·Pa^−0.5^, close to that obtained for a similar supported membrane of pure palladium. However, the manuscript does not mention the particular alloy composition achieved. In contrast to PdNi alloy, PdRu and PdPt [187] alloys showed long-term thermal stability at high temperatures. Membranes based on a PdRu thin film are prepared by co-deposition with really low contents on ruthenium (<2 wt %) [193], while the PdPt membranes are formed by alternating layers of Pd and Pt [85] with a final platinum load of around 25 wt %. El Hawa et al. prepared these types of alloys on modified porous stainless-steel with yttria-stabilized zirconia [187], reaching an average thickness of 6 μm. The H_2_ permeance achieved at 550 °C for membranes based on PdRu and PdPt alloys were 2.1 × 10^−3^ mol·s^−1^·m^−2^·Pa^−0.5^ and 1.39 × 10^−3^ mol·s^−1^m^−2^·Pa^−0.5^, respectively.

### 4.3. Ternary Alloys 

The formulation of ternary alloys has been also considered to combine simultaneously the improvements of each constituent [74]. However, published researches about preparation of multicomponent alloys by electroless plating is still scarce, being initiated only some years ago. First works suggest that particular compositions seems to reach an additional improvement on the membrane properties as compared to binary alloys, in terms of increasing hydrogen permeability and/or chemical resistance [56]. Alloying Pd simultaneously with two or more other metals (i.e., Ag, Cu, or Au) it is possible to improve not only the membrane permeability but also the mechanical and chemical resistances to sulphur poisons at the same time [41,201]. Additionally, the use of cheaper materials (i.e., Ag or Cu) in these formulations reduces the membrane cost [219,220,221,222]. On the other hand, copper and gold present higher melting points than silver, although lightly lower permeability of their binary alloys with palladium. Hence adding these metals on PdAg alloys to conform a ternary alloy could increase the thermal stability of the membrane [41,56,223].

**Figure 13:**
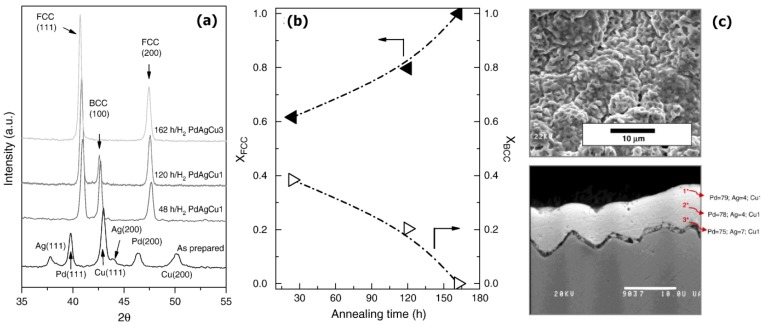
Ternary PdAgCu alloy formation: (**a**) XRD patterns after annealing up to 500 °C in H_2_ at different times, (**b**) Microstructure evolution with annealing time and (**c**) SEM images after annealing of both top surface and cross-section [224], with permission from © Elsevier.

Generally, the procedure to prepare these ternary alloys is similar to the previously described for obtaining binary alloys, including co-deposition or sequential electroless plating following by an annealing process at high temperature to finally obtain free-defects, homogeneous and continuous layers [74]. For instance, Tarditi et al. [224] presented the fabrication procedure of a PdAgCu ternary alloy on stainless-steel supports by consecutive deposition of palladium, silver and copper, in this order. They evidenced a hydrogen permeation flux of the ternary alloy about 70% higher than in case of considering a binary PdCu alloyed membrane with similar copper loading and average thickness of the selective layer. The annealing process for these membranes consists of a thermal treatment at 500 °C for 162 h, to obtain the FCC phase of the ternary alloy by XRD after the treatment. Figure 13 shows the evolution of the alloy during the annealing process.

In this work, no uniform distribution of metals was achieved, increasing silver content as going towards top surface in radial direction. Authors explain this effect by the lower surface tension of silver compared with this value in both palladium and copper, obtaining a surface segregation of silver after the annealing treatment [55]. 

Other researchers add gold to a previously prepared PdAg membrane to increase the resistance against H_2_S poison, in a similar way as using PdCu or PdAu binary alloys but with higher permeabilities derived from the presence of silver. For instance, Melendez et al. has lately published the preparation and testing of ternary PdAgAu alloys by incorporation of gold over a previously electroless plated PdAg membrane (using co-deposition of metals) on an asymmetric tubular Al_2_O_3_ support, with a thickness of 2.71 µm and final composition near to Pd_91.7_Ag_4.8_Au_3.5_ [41]. Prior to gold incorporation, the Pd-Ag layer was annealed at 550 °C for 4 h and the process was repeated again after incorporating gold. This membrane exhibited a H_2_ permeance of 4.71 × 10^−3^ mol·s^−1^·m^−2^·Pa^−0.5^ at 600 °C. The authors affirm that the H_2_/N_2_ ideal separation factor achieved with the ternary PdAgAu membrane was maintained relatively high after H_2_S exposure, in comparison with other binary PdAg membranes taken as reference. In fact, a Pd_96.1_Ag_3.9_ membrane suffered a decrease in hydrogen selectivity from 1308 to 18 after exposure to 9 ppm H_2_S for 15.25 h. However, the selectivity of a ternary alloy composition with composition like that of previously described, Pd_91.5_Ag_4.7_Au_3.8_, experienced a lower decrease in hydrogen selectivity, from 4115 to 800 after 9 ppm of H_2_S for 12.5 h. Moreover, ternary alloy membranes recovered original permeation rates in an 85% after sulphur exposure, whereas the hydrogen flux of PdAg membranes maintained below detectable values. 

Membranes formed by the alloy PdAuCu were also prepared by Tarditi et al. onto PSS disks with sequential electroless deposition of each metal [200]. First, the supports were cleaned and oxidized before incorporating a ZrO_2_ intermediate layer by using a vacuum-assisted dip-coating method. Palladium was deposited onto this modified support in two steps of 60 min, following by the incorporation of gold. Later, the membrane was rinsed and dried at 120 °C for 12 h prior to be activated again by conventional sensitization-activation process with SnCl_2_-PdCl_2_ solutions to allow for copper deposition on the top layer. Finally, in order to achieve an alloy with homogeneous composition, the membrane was annealed at 500 °C in H_2_ atmospheric pressure. After using this synthesis procedure, the best membrane was reached with 14 μm in thickness of the alloy composition Pd_69_Au_17_Cu_14_, obtaining a hydrogen permeance of 8.7 × 10^−9^ mol·s^−1^·m^−1^·Pa^−0.5^ at 400 °C and 50 kPa. The permeability value taken as reference in clean conditions were reduced around 55% after exposure to 100 ppm H_2_S/H_2_ at 400 °C for 24 h. However, authors affirmed that it was possible to recover around the 80% of the original permeation capacity of the membrane after testing with pure H_2_ at 400 °C.

Table 4 summarizes the most relevant information about Pd-alloy membranes detailed in this section, including information about metal incorporation, final thickness, alloy composition and annealing conditions, as well as used support and main permeation properties.

## 5. Concluding Remarks and Future Perspectives

This concluding section offers a perspective on current research in Pd-based membranes for H_2_ production processes, focused on dense supported membranes prepared by electroless plating, as well as future challenges need to be addressed for the real implementation of this technology in the industry. Supported membranes are preferred to reduce the thickness of the hydrogen selective layer and, consequently, the cost of the process. Moreover, the hydrogen permeation values, as well as thermal and mechanical resistances of the supported membranes, are increased comparing to unsupported membranes. Among the wide variety of materials that can be used as support, porous ceramic and 316L stainless-steel are the prevalent ones in recent researches. Particularly, it is expected that metal supports will prevail for industrial applications, above all at high temperatures, in which most devices are made in stainless-steel. Up to now, suitable ceramic-steel fitting for long operation times is not guaranteed. In this context, considering the wide pore size distribution and the high roughness of the metallic stainless-steel supports, it is usually required to modify its external surface with the aim to facilitate the incorporation of a thin selective palladium layer. One of the best alternatives is the incorporation of an intermediate layer, which simultaneously reduces the average pore size and roughness of the support top surface and avoids inter-diffusion problems. The main features to be considered to select the most suitable material used as interlayer are thermal compatibility between each component (support, barrier and selective layer) and membrane cost. Many of these intermediate layers are formed by metal oxides (i.e., CeO_2_, ZrO_2_ or SiO_2_ based materials) incorporated by dip-coating techniques. However, nowadays it has not been reached any prevalent solution and additional research in this issue is still required. Future directions are aimed to explore new materials or the combination of some of the already studied to achieve improved properties for the intermediate layer. 

Attending to the selective layer deposition, three main objectives are being followed: (i) reduction of the selective layer thickness (target: permeability increase and membrane cost reduction while ensuring complete absence of defects), (ii) reduction in the number of rejected membranes (target: to control the overall cost of the fabrication process) and (iii) preparation of Pd-based alloys (target: to improve permeation properties in real operation conditions, i.e., thermal cycles or presence of sulphur). To reach these objectives and to make easier the metal incorporation around the pores, several modifications of the conventional electroless plating method have been proposed in the last years, including the use of osmotic effect, vacuum or feeding both metal source and reducing agent from opposite sides of the support (ELP-PP). Finally, the use of binary Pd-based alloys to avoid hydrogen embrittlement of pure palladium or sulphur poisoning is also frequent, mainly adding specific amounts of silver, copper or gold. In the last years, an increasing number of alloy constituents is observed, trying to obtain membranes that gather particular benefits of different metals saving costs, i.e., hydrogen perm-selectivity of bulk palladium, increase of permeation rate provided by silver or sulphur resistance given by copper and gold. Here, it is very important to indicate the current limitation of possible metal constituents that can be incorporated by electroless plating and reproducibility between published results in terms of alloy composition, annealing conditions and hydrogen permeability. Thus, future trends go towards the combination of the most promising modifications for electroless plating with additional exploration of new Pd-based alloys with better properties in real industrial operation conditions (i.e., thermal cycling, presence of sulphur compounds, low hydrogen concentrations, etc.).

## Figures and Tables

**Table 1:** Usual inorganic commercial supports for Pd-based membrane preparation.

Company	Material	Geometry	Thickness (mm)	Porosity (%)	Pore Size (nm)
Mott	Stainless steel: 304 L, 316 L, 310, 347, 430	Disc, sheet, cup, tube	1–3		0.1–100 × 10^3^ (media grade)
	Hastelloy: C-22, C-276, X, N, B, B2				
	Inconel: 600, 625, 690				
GKN	Stainless steel: 304 L, 316 L, 904 L, 310	Disc, tube	1.5–3		0.1–200 × 10^3^ (media grade)
	Hastelloy: C-22, C-276, X				
	Inconel: 600, 625				
	Monel: 400				
	Bronze				
	Titanium				
Pall	Stainless steel: 304 L, 316 L, 310 SC	Cup, tube	- ^(a)^		>0.1 × 10^3 (a)^ (media grade)
	Hastelloy: X				
	Inconel: 600				
	Monel: 400				
	SiC/Al_2_O_3_				
	Mullite				
Inopor	α-Al_2_O_3_	Tube, multichannel tube	-	40–55	70–800
	TiO_2_			40–55	100–800
				30–55	5–30
				30–40	1
	ZrO_2_			40–55	110
				30–55	3
	γ-Al_2_O_3_			30–55	5–10
	SiO_2_			30–40	1
Tami	TiO_2_/ZrO_2_	Tube, multichannel tube	2		4.5 × 10^3 (b)^

^(a)^ On request, ^(b)^ Ultrafiltration grade with ZrO_2_ active layer (15 kg/mol).

**Table 2:** Inorganic commercial supports for supported Pd-based membrane preparation.

Support	Modification Alternative	Particular Details	Selective Layer	Tselective Layer (m)	Permeation Conditions		Permeation Capacity	H_2_ Separation Factor	Ref.
					T (°C)	P (kPa)			
PSS	Chemical treatment	HCl, 5 min.	Pd	20.0	350	100	3.11 × 10^−4 (a)^	5000	[110]
PSS	Chemical treatment	HCl-HNO_3_ mixture	Pd	5.0	450–550	100	3.24 × 10^−1^–4.34×10^−1 (c)^	n.a.	[111]
Ni	Chemical treatment	HCl	Pd	0.3	450	100	1.44 × 10^−1 (c)^	1600	[113]
Al_2_O_3_	Mechanical treatment	Sandpapers: #320, #500 and #800	Pd	0.5	n.a.	n.a.	n.a.	n.a.	[112]
Ni	Mechanical treatment	Sandpapers: #1200	PdCuNi	12.0	350–500	138–276	1.30 × 10^−7^–3.80 × 10^−7 (b)^	∞	[113]
PSS	Mechanical treatment	Ion shot penning	Pd	6.0	400	100	5.80 × 10^−2 (c)^	n.a.	[116]
PSS	Permanent Intermediate layer	CeO_2_ particles	Pd	13.0	550	200	2.75 × 10^−1 (c)^	∞	[122]
PSS	Permanent Intermediate layer	CeO_2_, sol-gel	PdCu	8.0	450	100	74.00 ^(a)^	2369	[123]
PSS	Permanent Intermediate layer	ZrO_2_, sol-gel		10.0	500	100	8.30 × 10^−2 (c)^	n.a.	[124]
PSS	Permanent Intermediate layer	ZrO_2_, sol-gel	PdCu	10.0	480	100	1.10 × 10^−7 (b)^	∞	[125]
PSS	Permanent Intermediate layer	ZrO_2_, sol-gel, vacuum assisted method	PdAu	10.0	400	100	1.10 × 10^−3 (a)^	>10,000	[93]
PSS	Permanent Intermediate layer	YSZ particles	Pd	27.7	350–450	30–400	4.50 × 10^−4 (a)^	∞	[70]
PSS	Permanent Intermediate layer	YSZ particles	Pd	13.8	350–450	0–250	4.10 × 10^−5^–4.10 × 10^−4 (a)^	∞	[78]
Hast X	Permanent Intermediate layer	YSZ–Al_2_O_3_/YSZ	PdAg	4.0–5.0	400–600	100	100.00 × 10^−8 (b)^	>200,000	[98]
PSS	Permanent Intermediate layer	γ-Al_2_O_3_, dip-coating	Pd	11.0	n.a.	n.a.	n.a.	n.a.	[130]
PSS	Permanent Intermediate layer	Graded Al_2_O_3_ particles	Pd	<5.0	500	n.a.	2.94 × 10^−3 (a)^	1124	[131]
PSS	Permanent Intermediate layer	SiO_2_ particles	PdCu	2.0	450	n.a.	8.37 × 10^−7 (d)^	70,000	[132]
PSS	Permanent Intermediate layer	Silicalite-1, sol-gel and dip-coating	Pd	5.0	350–450	50–250	1.42 × 10^−4 (a)^	∞	[62]
PSS	Permanent Intermediate layer	Zeolite NaA	Pd	19.0	450	50	1.10 × 10^−3 (a)^	608	[136]
PSS	Permanent Intermediate layer	Zeolite FAU-type	Pd	1.0	200	100	1.20 × 10^−4 (a)^	n.a.	[139]
Al_2_O_3_	Permanent Intermediate layer	Zeolite TS-1	Pd	2.0	350–450	50–500	1.48 × 10^−1 (c)^	148	[141]
PSS	Permanent Intermediate layer	Fe_2_O_3_-Cr_2_O_3_, oxidation in air (T = 600 °C)	Pd	33.0	300	n.a.	2.66 × 10^−4 (a)^	n.a.	[143]
PSS	Permanent Intermediate layer	Fe_2_O_3_-Cr_2_O_3_, oxidation in air (T = 600 °C)	Pd	19.0	n.a.	n.a.	n.a.	n.a.	[144]
PSS	Permanent Intermediate layer	Tungsten particles	PdCu	5.0–20.0	n.a.	n.a.	n.a.	n.a.	[47]
PSS	Temporary intermediate layer	Aluminum hydroxide gel/polymer	Pd	5.0	600	200	3.50 × 10^−3 (a)^	∞	[150]
Al_2_O_3_	Permanent Intermediate layer	Graphite-Clay (from 2B pencil)	Pd	5.0	450	100	3.10 × 10^−1 (c)^	3700	[151]
Al_2_O_3_	Permanent Intermediate layer	Pd(II)-modified bohamite sol	Pd	1.0	450	n.a.	2.23 × 10^−2^–1.07 ^(c)^	20–130	[152]
Al_2_O_3_	Permanent Intermediate layer	YSZ particles	Pd	5.0	150–500	150–400	0.10–0.60 ^(c)^	n.a.	[50]

Permeation capacity: ^(a)^ Permeance (mol·m^−2^·s^−1^·Pa^−0.5^), ^(b)^ Permeance (mol·m^−2^·s^−1^·Pa^−1^) or ^(c)^ Permeation flux (mol·m^−2^·s^−1^), n.a.: non available.

**Table 3:** Recent improvements on electroless plating to prepare supported Pd-based membranes.

ELP Improvement	Particular Details	Support	Support Modification	Tselective Layer (m)	Permeation Conditions		Permeation Capacity	H_2_ Separation Factor	Ref.
					T (°C)	P (kPa)			
Deposition around pores	Vacuum asisted-deposition	Al_2_O_3_	-	6.0	500	n.a.	8.78 × 10^−4 (a)^	3000	[176]
Deposition around pores	Vacuum asisted-deposition	Al_2_O_3_	Pd(II)-modified bohamite sol	1.0	450	n.a.	2.23 × 10^−2^–1.07 ^(b)^	20–130	[152]
Deposition around pores	Osmotic effect with aqueous sucrose solution	Vycor glass	-	1.6	n.a.	n.a.	n.a.	n.a.	[177]
Deposition around pores	Osmotic effect with aqueous sucrose solution	Vycor glass	-	2.5	n.a.	n.a.	n.a.	n.a.	[178]
Protecting selective layer	Pore- filled, vacuum asissted-deposition between two ZrO_2_ layers	Al_2_O_3_	YSZ particles	5.0	150–500	150–400	0.10–0.60 ^(b)^	n.a.	[50]
Reduction of carbon deposits	Free-EDTA baths	Al_2_O_3_	ZrO_2_	1.3	365	138	394.61 ^(a)^	n.a.	[181]
Reduction of carbon deposits	Free-EDTA baths	PSS	Al_2_O_3_	5.0	400	100	3.05·× 10^−3 (a)^	500	[182]
Increase film homogeneity	Support rotation	Al_2_O_3_	ZrO_2_	5.0	350–450	100–400	3.00·× 10^−3 (a)^	>400	[183]
Membrane repairing	Osmotic effect to close defects without thickness increase	PSS	-	10.0	425–475	68–136	2.00·10^−4 (b)^	400–1600	[179]
Membrane repairing	Point plating to close defects without thickness increase	α-Al_2_O_3_	γ-Al_2_O_3_	n.a.	500	100	7.20 × 10^−1^–8.50 × 10^−1 (b)^	n.a.	[184]
Reducing rejected membranes	ELP-PP. Pd-source and reducing agent from opposite sides of support	PSS	Fe_2_O_3_-Cr_2_O_3_	11.0–20.0	350–450	100–250	1.00 × 10^−4^–6.00 × 10^−4 (a)^	∞	[51]
Pd microstructure	Heat treatment at T > 640 °C	PSS	YSZ	4.9	600	82	2.40 × 10^−3 (a)^	200–2000	[42]

Permeation capacity: ^(a)^ Permeance (mol m^−2^·s^−1^·Pa^−0.5^) or ^(b)^ Permeation flux (mol·m^−2^·s^−1^). n.a.: non availabl.

**Table 4:** Recent advances on preparation of Pd-based alloys.

Alloy Type	Alloy Composition	ELP Metal Incorporation	Support	Support Modification	Tselective Layer (m)	Annealing	Permeation Conditions		Permeation Capacity	H_2_ Separation Factor	Sulfur Tolerance	Ref.
							T (°C)	P (kPa)				
Binary	Pd_75_Ag_25_	Sequential	Inconel	-	10.0	500 °C, 24 h	250–500	100	-	60–436	-	[211]
Binary	Pd_75_Ag_25_	Sequential	PSS	α-Al_2_O_3_/γ-Al_2_O_4_	20.0–26.0	500 °C	450	100	3.10 × 10^−4 (a)^	954	-	[202]
Binary	Pd_77_Ag_23_	Sequential	α-Al_2_O_3_/γ-Al_2_O_3_	-	2.3–2.5	500 °C, 800 h in H_2_	500	100	1.61–1.57 × 10^−2 (a)^	3770–5600	-	[53]
Binary	Pd_77_Ag_23_	Co-deposition	Al_2_O_3_	-	3.2	500 °C, 2 h in N_2_	400	100	3.10 × 10^−6 (a)^	8000–10,000	-	[95]
Binary	PdAg	Co-deposition	Hast X	YSZ–Al_2_O_3_	4.0–5.0	n.a.	4–600	100	100.00 × 10^−8 (b)^	>200,000	-	[192]
Binary	Pd_81_Cu_19_	Sequential	Al_2_O_3_	-	5.0	500 °C, 48 h in N_2_	400	100	1.20 × 10^−3 (a)^	1194	Yes (35 ppm)	[54]
Binary	Pd_60_Cu_40_	Sequential	α-Al_2_O_3_/γ-Al_2_O_3_; α-Al_2_O_3_/ZrO_2_	-	11.0	H_2_ atmosphere	450	345	0.80 ^(b)^	1150	Yes	[84]
Binary	Pd_62_Cu_38_	Sequential	PSS	CeO_2_	8.0	480 °C, 6 h in H_2_	450	100	74.00 ^(a)^	2369	Yes	[123]
Binary	Pd_90_Au_10_	Sequential, galvanic displacement	PSS	Oxidation in air (700 °C, 12 h)	<15.0	500 °C, 48 h in H_2_	3–500	100	9.35 × 10^−4 (a)^	∞	Yes (54.8 ppm)	[94]
Binary	Pd_91_Au_9_	Sequential, galvanic displacement	PSS	ZrO_2_	10.0	500 °C in H_2_	400	100	1.10 × 10^−3 (a)^	>10,000	Yes (54.8 ppm)	[93]
Binary	Pd_x_Ni_y_	Sequential	α-Al_2_O_3_	-	7.0	n.a.	500	20–120	2.74 × 10^−3 (a)^	640	-	[218]
Binary	Pd_98_Ru_2_	Co-deposition	PSS	YSZ	6.0	n.a.	550	n.a.	2.10 × 10^−3 (a)^	1860	-	[187]
Binary	P_75_Pt_25_	Co-deposition	PSS	YSZ	6.0	n.a.	550	n.a.	1.39 × 10^−4 (a)^	1590	-	[187]
Ternary	Pd_x_Ag_y_Cu_z_	Sequential	PSS	Oxidation in air (500 °C, 12 h)	24.0–27.0	500 °C, 162 h	3–450	10–100	1.70–2.10 × 10^−4 (a)^	300–10,000	n.a.	[224]
Ternary	Pd_91.7_Ag_4.8_Au_3.5_	Co-deposition/Sequential	α-Al_2_O_3_/γ-Al_2_O_3_	-	2.7	550 °C, 8 h	600	n.a.	4.71 × 10^−3 (a)^	n.a.	Yes (9 ppm)	[41]
Ternary	Pd_91.5_Ag_4.7_Au_3.8_	Co-deposition/Sequential	α-Al_2_O_3_/γ-Al_2_O_4_	-	2.7	550 °C, 8 h	600	n.a.	2.32 × 10^−3 (a)^	4115–793	Yes (9 ppm)	[41]
Ternary	Pd_69_Au_17_Cu_14_	Sequential	PSS	ZrO_2_	14.0	500 °C in H_2_	400	50	6.20 × 10^−4 (a)^	n.a.	Yes (100 ppm)	[200]

Permeation capacity: ^(a)^ Permeance (mol·m^−2^·s^−1^·Pa^−0.5^) or ^(b)^ Permeation flux (mol·m^−2^·s^−1^). n.a.: non available.
